# Necrosis of the Superior and Inferior Maxilla

**Published:** 1852-10

**Authors:** 


*Necrosis of the Superior and Inferior Maxilla.*—There is a very curious fact that appears to remain unnoticed as far as our observation extends, viz. that in all of the bones of the trunk, limbs, &c., suffering under caries and necrosis, nature makes a constant effort to recuperate by granulation and fresh deposition, but with the maxilla there is no evidence of their possessing this power in the slightest degree, partaking as it were of the character of the teeth in this respect.* The lower jaw in young persons occasionally perishes without any previous derangement of health, local injury, or other apparent cause, its death preceded only by an aching in the bone, but such cases occur almost invariably in early life from the age of four to twenty years. There is sometimes found after death of the jaw from necrosis a deposition around the bone resembling, to a great extent, calculi, and which Stanley describes as a gray pumice-like osseous substance, deposited from the inflamed periosteum, and he regards it as a morbid product from a diseased tissue, being wholly disorganized matter. Dr. Heyfelder, of Berlin, published a series of very interesting cases of necrosis, occurring from the inhalation of *phosphorous acid vapor*, given off in factories for lucifer-matches, affecting either or both jaws, depending upon the condition of the general health, and corroborating the above opinion that the loss of these bones is never followed by the slightest reproduc-

* There is a case recorded in the Chirurgical Journal, vol. ii, of the reproduction of the whole ramus of the jaw with its condyles and coronoid process, after the operation of extraction had been performed by Desault. If this be well authenticated, it is an isolated case, and has no bearing upon the general law, as it is generally admitted that the periosteum of a bone is the reproductive agent; in such a case this tissue must have been wholly destroyed, together with a great deterioration of the surrounding tissues.

tion. It is stated that necrosis of the jaws is most frequently caused from the use of calomel, administered in fevers and from severe inflammation of the gums from carious, dead or injured teeth, or from a large accumulation of tartar.

